# Supplementary material for: Atypical local and global biological motion perception in children with attention deficit hyperactivity disorder
Source: eLife. 2024 Jul 2;12:RP90313. doi: 10.7554/eLife.90313 (PMC11219041; doi:10.7554/eLife.90313)
Supplement: Peer review history

# Editor report: eLife assessment

**DOI:** 10.7554/eLife.90313.5.sa0

**Authors:** Clare Press

**Affiliations:** University College London United Kingdom

**Keywords:** Important, Solid

The authors use point light displays to measure biological motion (BM) perception in children (mean = 9 years) with and without ADHD, and relate it to IQ, social responsiveness scale (SRS) scores and age. They report that children with ADHD were worse at all three BM tasks, but that those tasks loading more heavily on local processing relate to social interaction skills and those loading on global processing relate to age. There are still some elements of the results that need clarification with future work, but nevertheless, the **important** and **solid** findings extend our limited knowledge of BM perception in ADHD, as well as biological motion processing mechanisms in general.

# Referee report: Reviewer #2 (Public Review)

**DOI:** 10.7554/eLife.90313.5.sa1

Summary:

Tian et al. aimed to assess differences in biological motion (BM) perception between children with and without ADHD, as well as relationships to indices of social functioning and possible predictors of BM perception (including demographics, reasoning ability and inattention). In their study, children with ADHD showed poorer performance relative to typically developing children in three tasks measuring local, global, and general BM perception. The authors further observed that across the whole sample, performance in all three BM tasks was negatively correlated with scores on the social responsiveness scale (SRS), whereas within groups a significant relationship to SRS scores was only observed in the ADHD group and for the local BM task. Local and global BM perception showed a dissociation in that global BM processing was predicted by age, while local BM perception was not. Finally, general (local & global combined) BM processing was predicted by age and global BM processing, while reasoning ability mediated the effect of inattention on BM processing.

Strengths:

Overall, the manuscript is presented in a clear fashion and methods and materials are presented with sufficient detail so the study could be reproduced by independent researchers. The study uses an innovative, albeit not novel, paradigm to investigate two independent processes underlying BM perception. The results are novel and have the potential to have wide-reaching impact on multiple fields.

Weaknesses:

The manuscript has improved in clarity and conceptual and methodological considerations in response to the last review. However, the reported results still provide incomplete support for the claims the authors make in the paper, due to differences between correlations not passing significance thresholds.

# Referee report: Reviewer #3 (Public Review)

**DOI:** 10.7554/eLife.90313.5.sa2

The authors presented point light displays of human walkers to children (mean = 9 years) with and without ADHD to compare their biological motion perception abilities, and relate them to IQ, social responsiveness scale (SRS) scores and age. They report that children with ADHD were worse at all three biological motion tasks, but that those loading more heavily on local processing related to social interaction skills and global processing to age. The valuable and solid findings are informative for understanding this complex condition, as well as biological motion processing mechanisms in general. However, the correlations present a pattern that needs further examination in future studies because many of the differences between correlations are not significant.

Strengths:

The authors present differences between ADHD and TD children in biological motion processing, and this question has not received as much attention as equivalent processing capabilities in autism. They use a task that appears well controlled. They raise some interesting mechanistic possibilities for differences in local and global motion processing, which are distinctions worth exploring. The group differences will therefore be of interest to those studying ADHD, as well as other developmental conditions, and those examining biological motion processing mechanisms in general.

Weaknesses:

The data are not strong enough to support claims about differences between global and lobal processing wrt social communication skills and age. The mechanistic possibilities for why these abilities may dissociate in such a way are interesting, but the crucial tests of differences between correlations do not present a clear picture. Further empirical work would be needed to test this further. Specifics:

The authors state frequently that it was the local BM task that related to social communication skills (SRS) and not the global tasks. However, the results section shows a correlation between SRS and all three tasks. The only difference is that when looking specifically within the ADHD group, the correlation is only significant for the local task. The supplementary materials demonstrate that tests of differences between correlations present an incomplete picture. Currently they have small samples for correlations, so this is unsurprising.

Theoretical assumptions. The authors make some statements about local vs global biological motion processing that may have been made in previous studies, but would appear controversial and not definitive. E.g., that local BM processing does not improve with age.

# Author comment: Author response

**DOI:** 10.7554/eLife.90313.5.sa3

**Authors:** Junbin Tian, Fang Yang, Ying Wang, Li Wang, Ning Wang, Yi Jiang, Li Yang

**Affiliations:** Peking University Sixth Hospital Beijing China; University of Chinese Academy of Sciences Beijing China; Chinese Academy of Sciences Beijing China; Chinese Academy of Sciences Beijing China; Peking University Sixth Hospital 北京 China; Chinese Academy of Sciences Beijing China; Peking University Sixth Hospital Beijing China

The following is the authors’ response to the previous reviews.

**eLife assessment**

The authors use point light displays to measure biological motion (BM) perception in children (mean = 9 years) with and without ADHD, and relate it to IQ, social responsiveness scale (SRS) scores and age. They report that children with ADHD were worse at all three BM tasks, but that those tasks loading more heavily on local processing relate to social interaction skills and those loading on global processing relate to age. There are still some elements of the results that are unclear, but nevertheless, the important and solid findings extend our limited knowledge of BM perception in ADHD, as well as biological motion processing mechanisms in general.

We thank the editors and reviewers for their valuable feedback and constructive comments. In the revised manuscript, we have incorporated all statistics for the models and also provided detailed analytical evidence about the distinct contributions of local and global BM processing. We hope these clarifications could enhance the robustness of our conclusions.

**Public Reviews:**

**Reviewer #2 (Public Review):**

Summary:

Tian et al. aimed to assess differences in biological motion (BM) perception between children with and without ADHD, as well as relationships to indices of social functioning and possible predictors of BM perception (including demographics, reasoning ability and inattention). In their study, children with ADHD showed poorer performance relative to typically developing children in three tasks measuring local, global, and general BM perception. The authors further observed that across the whole sample, performance in all three BM tasks was negatively correlated with scores on the social responsiveness scale (SRS), whereas within groups a significant relationship to SRS scores was only observed in the ADHD group and for the local BM task. Local and global BM perception showed a dissociation in that global BM processing was predicted by age, while local BM perception was not. Finally, general (local & global combined) BM processing was predicted by age and global BM processing, while reasoning ability mediated the effect of inattention on BM processing.

Strengths:

Overall, the manuscript is presented in a clear fashion and methods and materials are presented with sufficient detail so the study could be reproduced by independent researchers. The study uses an innovative, albeit not novel, paradigm to investigate two independent processes underlying BM perception. The results are novel and have the potential to have wide-reaching impact on multiple fields.

We appreciate the your positive feedback very much.

Weaknesses:

The manuscript has improved in clarity and conceptual and methodological considerations in response to the last review. However, the reported results still provide incomplete support for the claims the authors make in the paper.

In relation to other reviewers' earlier comments, the model notation used is still not consistent and model results are reported incompletely, which make it difficult to gain a full picture of the data and how they support the authors' secondary claims. For instance, across the models in the supplementary materials, ß coefficients are only reported selectively which makes it difficult to assess the model as a whole. Furthermore, different terms (task 1, task 2 vs. BM-Local, BM-global) are used to refer to the same levels of a variable, and it is unclear which levels of a dummy variable correspond to which task, making it overall very difficult to comprehend the modelling procedure.

Thanks for pointing out these issues. In the revised version, we have unified the terminology by consistently referring to task types as BM-Local, BM-Global, BM-General. Additionally, we have provided clarification on the interpretation of dummy variables in relation to model construction. Furthermore, we corrected the model results and included all statistics in Table S1, S2, and S3. For more detailed information, please refer to the response to your Recommendations for the authors.

**Reviewer #3 (Public Review):**

The authors presented point light displays of human walkers to children (mean = 9 years) with and without ADHD to compare their biological motion perception abilities, and relate them to IQ, social responsiveness scale (SRS) scores and age. They report that children with ADHD were worse at all three biological motion tasks, but that those loading more heavily on local processing related to social interaction skills and global processing to age. The valuable and solid findings are informative for understanding this complex condition, as well as biological motion processing mechanisms in general. However, the correlations present a pattern that needs further examination in future studies because many of the differences between correlations are not significant.

Strengths:

The authors present differences between ADHD and TD children in biological motion processing, and this question has not received as much attention as equivalent processing capabilities in autism. They use a task that appears well controlled. They raise some interesting mechanistic possibilities for differences in local and global motion processing, which are distinctions worth exploring. The group differences will therefore be of interest to those studying ADHD, as well as other developmental conditions, and those examining biological motion processing mechanisms in general.

Thanks for this positive assessment of our work.

Weaknesses:

The data are not strong enough to support claims about differences between global and lobal processing wrt social communication skills and age. The mechanistic possibilities for why these abilities may dissociate in such a way are interesting, but the crucial tests of differences between correlations do not present a clear picture. Further empirical work would be needed to test this further. Specifics:

The authors state frequently that it was the local BM task that related to social communication skills (SRS) and not the global tasks. However, the results section shows a correlation between SRS and all three tasks. The only difference is that when looking specifically within the ADHD group, the correlation is only significant for the local task. The supplementary materials demonstrate that tests of differences between correlations present an incomplete picture. Currently they have small samples for correlations, so this is unsurprising.

We apologize for not clarifying these points earlier. We did identify correlations between performance on all BM tasks and SRS scores. However, it is noteworthy that this finding is not unexpected, given the significant distinctions in SRS scores between TD and ADHD children, alongside their marked differences in all BM tasks. Correlation analyses involving data from both groups may reflect group differences. To elucidate the relationship between social ability impairment and diminished BM processing in children with ADHD, we conducted additional subgroup analyses and found correlations only in the BM-local task. To further support the specificity of this correlation, we compared the differences in coefficients. We revised our modelling procedure for testing differences between correlations in supplementary materials and presented all models statistics in Table S2, S3. Discrepancies in these coefficients, which exclude the influence of differences between groups, suggest that social factors specifically influence the performance of the BM-Local task in children with ADHD. We acknowledge that the analysis for differences between correlations is based on a relative small sample size and provided modest interpretation in discussion. Future studies will aim to increase the sample size to validate our findings.

Theoretical assumptions. The authors make some statements about local vs global biological motion processing that may have been made in previous studies, but would appear controversial and not definitive. E.g., that local BM processing does not improve with age and is uninfluenced by attention.

Thanks for your comment. To the best of our knowledge, there have been fewer developmental studies conducted on local BM processing compared to global BM processing. Our study is the first one to directly explore the relationship between local BM processing and age. Additionally, we used QbInattention to evaluate sustained attention function (considered as “top-down” attention) and examined its correlation with local BM processing. Some indirect evidence supported that the ability to process local BM cues remained stable and was unaffected by top-down attention. For example, local BM processing did not show a learning trend (Chang 2009) and was linked to the activation of subcortical regions (Hirai 2020). Research has demonstrated that local BM cues can convey information about walking direction without participants’ explicit attention or recognition (Chang 2009, Hirai 2011, Thompson 2007, Wang 2010), indicating the involvement of “bottom-up” processing (Hirai 2020, Troje 2023). Consistent with previous findings, we did not find significant correlation between local BM processing and age or QbInattention. We acknowledge that the statement such as “local BM processing does not improve with age and is uninfluenced by attention” should be approached with cautions. Therefore, we interpreted our results carefully:

“Once a living creature is detected, an agent (i.e., is it a human?) can be recognised by a coherent, articulated body structure that is perceptually organised based on its motions (i.e., local BM cues)71. This involves top-down processing and probably requires attention25,72, particularly in the presence of competing information26. Our findings are consistent with those of previous studies on the cortical processing of BM73, as we found that the severity of inattention in children with ADHD was negatively correlated with their performance in global BM processing, whereas this significant correlation was not found in local BM processing, which may involve bottom-up processing61,65 and might not need participants’ explicit attention21,23,74,75. However, further studies are needed to verify this hypothesis.” (lines 461-470)

**Recommendations for the authors:**

**Reviewer #2 (Recommendations For The Authors):**

Supplementary materials: For all reported results, I suggest the authors use consistent model notation with complete reporting of all statistics in line with common conventions (ideally tables reporting beta values, error terms and confidence intervals for all model predictors, as well as R squared values). In particular the beta values for the reference category are needed to be able to fully interpret the beta values for the reported contrasts.

We appreciate the your suggestion. In the newly revised manuscript, we reported all statistics including beta values, error terms and confidence intervals for all model predictors, and R squared values. These detailed statistics can be found in Table S1, S2 and S3. We hope this additional information will offer readers a more comprehensive understanding of our study.

Please also address the following inconsistencies:

- At least when reporting the model results, the same term should be used when refering to task type (either task 1/2/3/ or local/global/general BM).

Thank the your for this feedback. We use the same term (BM-Local/Global/General) to refer to task type in the whole text.

- Second linear model in the Supplementary Materials: The authors state that the results suggest that the correlation between SRS and task 1 is greater than that between task 2 and SRS scores. First of all, to be able to support this claim the authors need to provide the coefficient for task 1 (which, if task 1 is the reference variable should be ß1). Second, as I currently understand the reported model results, the fact that ß4 (representing the difference in relationship to SRS scores between task 2 and task 1; the authors refer to ß3 here although I assume they mean ß4) is negative and shows a trend towards significance would actually mean the relationship between BM processing accuracy and SRS scores is more negative for task 2 relative to task 1 and not, as the authors state, that the correlation with SRS scores is greater for task 1. I realise this contradicts the individual r values and scatter plots and hope the authors can clarify the model results.

We thank you for pointing out these issues. For the second linear model (Model 4 in revised manuscript), we reported the coefficients for all predictors and model summaries including the coefficient for task 1 (ß1). In addition, we have made correction to the model results. The values of ß4 (representing the difference in relationship to SRS scores between BM-Global and BM-Local) and ß5 (representing the difference in relationship to SRS scores between BM-General and BM-Local) were positive and showed a trend towards significance, indicating that the correlations with SRS total score were more negative for BM-Local relative to BM-Global and BM-General:

“A general linear model was constructed (Table S2, Model 4): SRS = β0 + β1 * ACC + β2 * D1 + β3 * D2 + β4 * (ACC * D1) + β5 * (ACC * D2). If the effect of the interaction term (i.e., β4 or β5 ) is statistically significant, it indicates a difference in correlations with SRS total score between BM-Local and BM-Global (or BM-General). The results suggested trends where the correlations with SRS total score were more negative for BM-Local relative to BM-Global (standardized β4 = 0.580 p = 0.074) and BM-General (standardized β5 = 0.550 p = 0.073).” (lines SI 36-42)

- Third linear model in the Supplementary Materials: In the dummy variable representing task, when local BM is the reference level, which task is represented by d1 and d2, respectively? If I understand the authors' procedure correctly, d1 should represent the difference between local and global BM and d2 the difference between local and general BM. If this is true, ß4 should code for the difference between local and global BM and not, as stated by the authors, for the difference between local and general BM. Also, what is d3?

Thank you for pointing out this issue. We corrected and clarified the results of third model (Model 5 in revised manuscript) in the revised version and pointed out what is represented by d1 (D1) and d2 (D2), respectively:

“We recoded task types into two dummy variables, D1 and D2, using BM-Local as a reference. The coefficient of D1 represents the difference in relationship to age between BM-Local and BM-Global, and the coefficient of D2 represents the difference in relationship to age between BM-Local and BM-General. The following model was created for each group (Table S3, Model 5-6): ACC = β0 + β1 * age + β2 * D1 + β3 * D2 + β4 * (age * D1) + β5 * (age * D2). If the effect of the interaction term (i.e., β4 or β5) is statistically significant, it indicates a difference in the effect of age on ACC between BM-Local and BM-Global (or BM-General). In the ADHD group, we observed a significant difference in the effect of age on ACC between BM-Local and BM-General (standardized β5 = 0.462, p < 0.001) and marginally significant differences in the effect of age on ACC between BM-Local and BM-Global (standardized β4 = 0.228, p = 0.073).” (lines SI 47-57)
